# Genomic Insights into Winter Wheat Breeding for Severely Cold Climates

**DOI:** 10.3390/ijms27031568

**Published:** 2026-02-05

**Authors:** Demissew Sertse, Wubishet A. Bekele, Curt A. McCartney

**Affiliations:** 1 Department of Plant Science, University of Manitoba, Winnipeg, MB R3T 2N2, Canada; 2 Ottawa Research and Development Centre, Agriculture and Agri-Food Canada, Ottawa, ON K1A 0C6, Canada

**Keywords:** winter wheat, genome scanning, extreme cold winter climate, genomic signature

## Abstract

Wheat is one of the world’s most important crops, cultivated across diverse ecogeographic zones on more than ~245 million hectares annually. Classified by vernalization requirement into spring, facultative, or winter types, the latter typically achieves higher yields due to its extended growing season, reaching ~18 t ha^−1^ and 9–10 t ha^−1^ as a national average for Western European countries such as Germany, France, and England, compared with the global average of barely above 3 t ha^−1^. Despite this potential, winter wheat is largely confined to regions with relatively mild winters, while vast temperate zones with extremely cold winters rely on spring wheat. Breeding has traditionally targeted the *vernalization–C-repeat Binding Factor* (*VRN–CBF*) pathway, which confers tolerance to moderately severe winters but is insufficient for extreme cold, implying the need for additional layers of adaptive mechanisms. Using multiple genotypic datasets, we identified genomic regions underlying low-temperature tolerance. Genome- and chromosome-wide scans revealed strong differentiation on chromosome 5A (526–703 Mb), overlapping the *VRN–CBF* loci. SNP-level *FST* analysis between spring and winter cultivars highlighted the *VRN-A1* (586–588 Mb) region and a locus spanning 549 and 559 Mb on chromosome 6A. Further comparisons between winter accessions adapted to extreme cold (≤−12 °C) and mild winters (>0 °C) revealed a differentiated region on chromosome 3B (561–564 Mb) harbouring two key genes conferring CBF-independent cold tolerance, *TRAESCS3B02G351100* and *TRAESCS3B02G354000*, encoding diacylglycerol kinase1 (DGK1) and peroxidase 56 (PRX56), respectively. These findings underscore alternative pathways in shaping cold adaptation, highlighting the need to broaden breeding strategies for extreme environments. We further detected a pronounced haplotype divergence between Chinese and U.S. winter cultivars reflecting distinct breeding trajectories; notably, China, where ~90% of wheat production is of the winter type, achieves national yields >5 t ha^−1^, compared with ~3 t ha^−1^ in the United States, where over 70% of production is winter wheat. This contrast suggests that the haplotypes enriched in Chinese winter cultivars could represent valuable resources for enhancing winter wheat performance in other regions with comparable environments.

## 1. Introduction

Wheat stands among the world’s most important staple crops, currently ranking third in global production volume with an average annual harvest of ~7.2 million metric tons, following closely behind maize (~8.3 million tons) and rice (~7.4 million tons), according to FAOSTAT (2023) [1]. However, in terms of land use, wheat leads all cereals, occupying the largest cultivated area worldwide, with an average of ~245 million hectares under production each year over the past decade [1].

The crop adapts to diverse environments, thriving across an extensive spectrum of agroecological zones worldwide. From the high-latitude temperate climates of the Northern Hemisphere to the warm, rainfed lowlands of the tropics, wheat has proven its resilience and versatility under diverse environmental conditions. This broad ecological amplitude is a testament to the crop’s vast genetic diversity, its enduring role in ensuring global food security, and the profound legacy of human intervention and innovation since its initial domestication [2].

Over the course of ~10,000 years of cultivation, wheat has undergone significant evolutionary and agronomic transformations [3]. Through human-guided breeding and geographic dispersion, it has differentiated into multiple ecotypes, each adapted to specific climatic and soil conditions [3,4]. Furthermore, it has diversified into a wide array of end-use types, from bread wheat and durum wheat to more regionally specialized forms, each aligned with cultural preferences and food systems [5].

Wheat has evolved mainly into two distinct growth habits: spring wheat, suited to shorter growing seasons or regions with warm climates, and winter wheat, which requires vernalization and overwinters under cold and snowy conditions. Additionally, some facultative lines possess intermediate adaptability, allowing them to thrive under both winter and spring growing conditions.

Beyond their growth habit differences, winter and spring also differ markedly in yield potential, with winter wheat generally outyielding its spring counterpart. The winter wheat yield advantage is largely attributed to its longer growing period, allowing it to have a well-established root system that makes the crop more efficiently use soil nutrients and moisture and accumulate greater biomass, enhancing stress resilience, grain filling, and hence yield. The winter wheat type scored a yield record of ~18 tons/ha in the UK [6,7], which is over five times the current global average, ~3.4 tons/ha [1].

Despite the well-documented yield advantage of winter wheat over spring wheat, vast tracts of land across major agricultural regions, including western Canada (Prairies) and the northern United States, remain predominantly reliant on spring wheat cultivation. This reliance persists even in areas where the agroecological potential favors winter wheat production if strong research efforts are in place to develop resilient varieties and optimize agronomic conditions.

Transitioning from spring to winter wheat in these regions holds immense promise. Not only could such a shift substantially enhance local yields, but it could also meaningfully contribute to global wheat output, an increasingly urgent priority in light of the world’s growing population, which is projected to approach 10 billion by the middle of this century. Hence, enhancing winter wheat production in cold climate regions would serve as a strategic move toward securing the global food supply and meeting future demands.

However, several formidable challenges have hindered the broader adoption of winter wheat in these regions. The two major challenges are the harsh winter conditions characterized by prolonged exposure to extremely low temperatures and, more critically, the occurrence of intermittent freeze–thaw cycles. These fluctuating winter temperatures can prematurely break the dormancy of overwintering wheat, leaving the plants vulnerable to intracellular ice crystal formation, a phenomenon that disrupts cell membranes and results in widespread winterkill and damage from snow mold.

Overcoming these climatic obstacles requires concerted investment in breeding winter wheat varieties with enhanced tolerance to extreme cold and resilience to fluctuating temperatures, resulting in multiple freeze–thaw cycles over the winter months. It also necessitates innovative agronomic practices and predictive modelling to better optimize growing fields and synchronize planting and dormancy cycles. Unlocking the potential of winter wheat in these extreme-cold winter regions could represent one of the most impactful strides toward sustainable global wheat production in the coming decades.

The convergence of vast genomic and environmental datasets, housed in public repositories and enriched continually through rapid, cost-effective data generation, has accompanied a new era for plant breeding and agricultural innovation. Recent technological advancements in high-throughput genotyping and computational tools, including artificial intelligence’s potential to revolutionize our ability to integrate such diverse datasets and extract meaningful patterns from complex data landscapes, enable breeders to precisely identify and trace germplasm carrying desirable traits from expansive global gene pools, including landraces, wild relatives, and elite cultivars.

In this study we analyze publicly available genetic and environmental data to identify genomic regions potentially associated with winter hardiness and identify candidate accessions based on the alleles they carry at identified potential loci.

## 2. Results

### 2.1. Cold Tolerance-Related Loci and Their Candidate Genes

**Figure 1:**
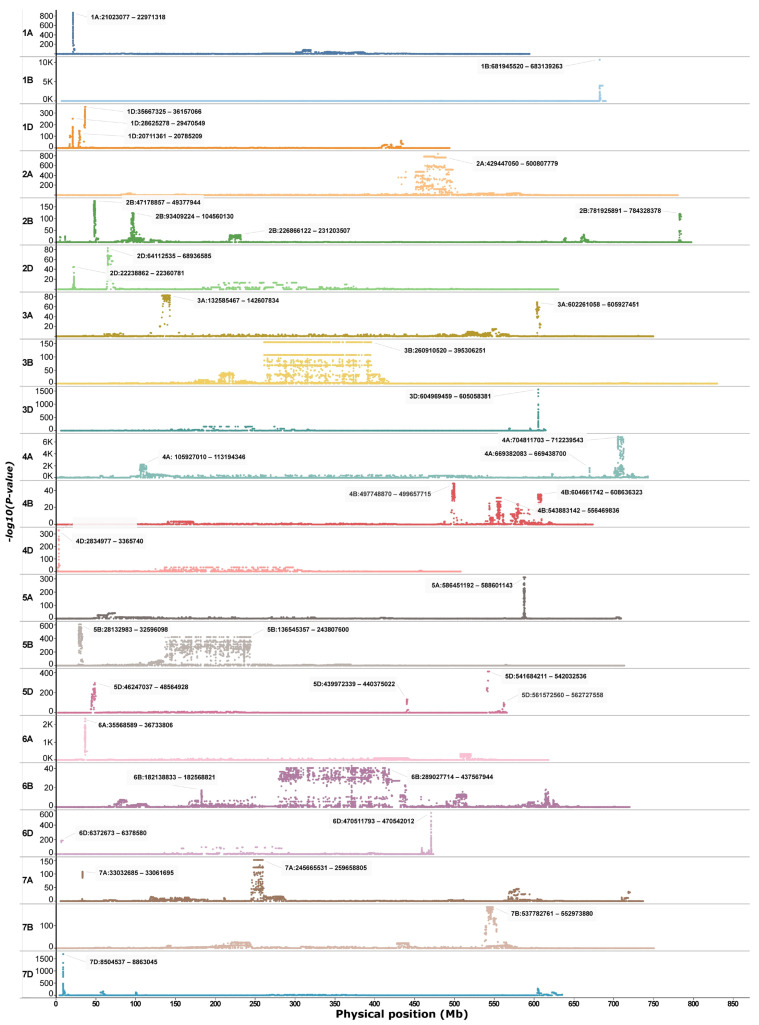
Genetic signatures identified through a principal component-based genome scan analysis of the WGIPK dataset.

Genome scans of the winter-dominated IPK dataset revealed several uniquely differentiated regions across the genome (Figure 1). Excluding chromosome 3B, which exhibited an extensive differentiated region spanning approximately 260 to 400 Mb (Figure 1), a total of 80 genes associated with cold tolerance were identified across various peak regions in the winter-dominant IPK scan. These genes were distributed over 15 chromosomes (Table S1), with peak regions on chromosome 2B contributing more than 15 genes (Table S1). Additionally, 13 genes linked to climatic variables during the coldest quarter of the year, including the minimum temperature, were detected; of these, only a single gene on chromosome 4A overlapped with previously reported cold tolerance loci (Table S1).

**Figure 2:**
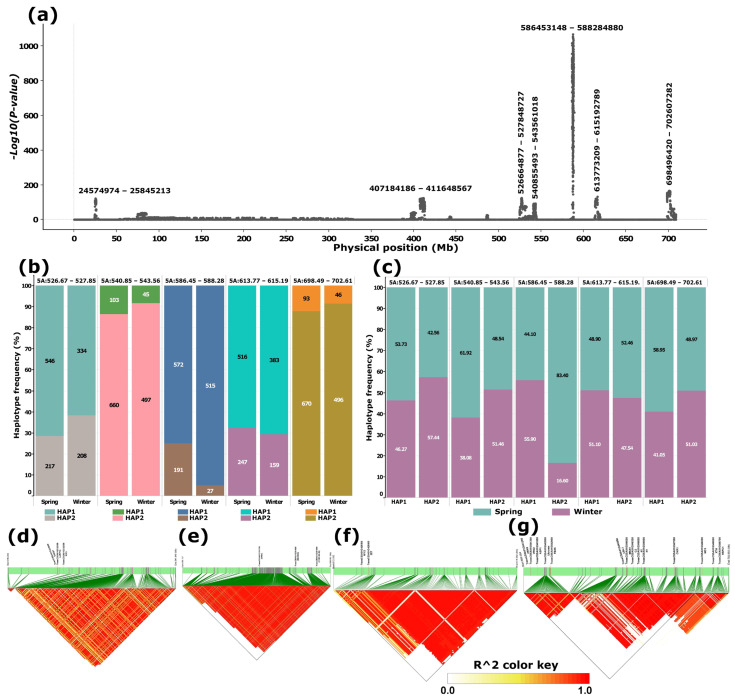
Principal component-based genome scan of chromosome 5A and linkage disequilibrium (LD) blocks for selected differentiated loci. (**a**) Manhattan plot illustrating genomic regions exhibiting significant differentiation; labels above major peaks denote the span of each differentiated region (bp). (**b**) Haplotype frequency distributions at selected loci in spring and winter wheat types. (**c**) Proportion of spring and winter wheat types represented within each haplotype at the selected loci. (**d**–**g**) LD block structures corresponding to the four most prominent peaks, shown in genomic order. Panel (**e**) highlights the LD blocks associated with the most conspicuous peak, located between approximately 586–589 Mb. Candidate genes positioned above the green bars represent potential targets for breeding or selection.

A targeted genome scan of chromosome 5A using the merged dataset, comprising three whole-genome datasets (WGIPK, WGWat, and WGCHNUS), revealed a conspicuously differentiated region between 586 and 589 Mb consistent with the WGIPK genome-wide scan (Figure 1 and Figure 2). The chromosome 5A scan of the merged dataset identified an additional six other differentiated regions, four of which were located within the 130 Mb range of the most differentiated region (586–589 Mb) (Figure 2a). Each of these five differentiated regions, including the 586–589 Mb, predominantly harboured two haplotypes, one carried by at least over 800, and the other by over 200, accessions (Figure 2b). Noticeable haplotype frequency variations were observed between spring and winter wheat types in regions spanning 526-528 Mb, and 586–589 Mb, where the minor haplotypes showed a relatively higher frequency in winter wheat for the former, andin spring wheat for the latter (Figure 2b). Further distribution analyses showed that, in the most strongly differentiated region (586–589 Mb), the minor haplotype (HAP2) occurred at a frequency exceeding 80% in spring wheat (Figure 2c). 

SNPs within each of the regions were in a moderate to strong linkage disequilibrium (Figure 2d–g). Of the last four regions, including the most differentiated segment (586–589 Mb), three of them harboured cold tolerance genes. Notably, the 586–589 Mb region harboured two cold tolerance-related genes, *TRAESCS5A02G391700* (*VRN1*) and *TRAESCS5A02G391800* (*MADS34*), alongside *TRAESCS5A02G391900* (*CYB5#6/B5#6*), where all three appeared in a strong linkage disequilibrium (LD) block (Figure 2e). The last peak (698–703 Mb) harboured a relatively higher number of these genes involved in cold tolerance, including two *VRN2* genes, *TRAESCS5A02G541200* and *TRAESCS5A02G541300*. This region appeared to be in three LD blocks, where the first LD block comprised the two *VRN2* genes. The region also harboured two FLOWERING LOCUS T (FT) genes: *TRAESCS5A02G546800* and *TRAESCS5A02G546900* in the second LD block and Xyloglucan endotransglucosylase/hydrolase (XTH) and Heat Shock Factor (HSFC1) in the third LD block (Figure 2g).

**Figure 3:**
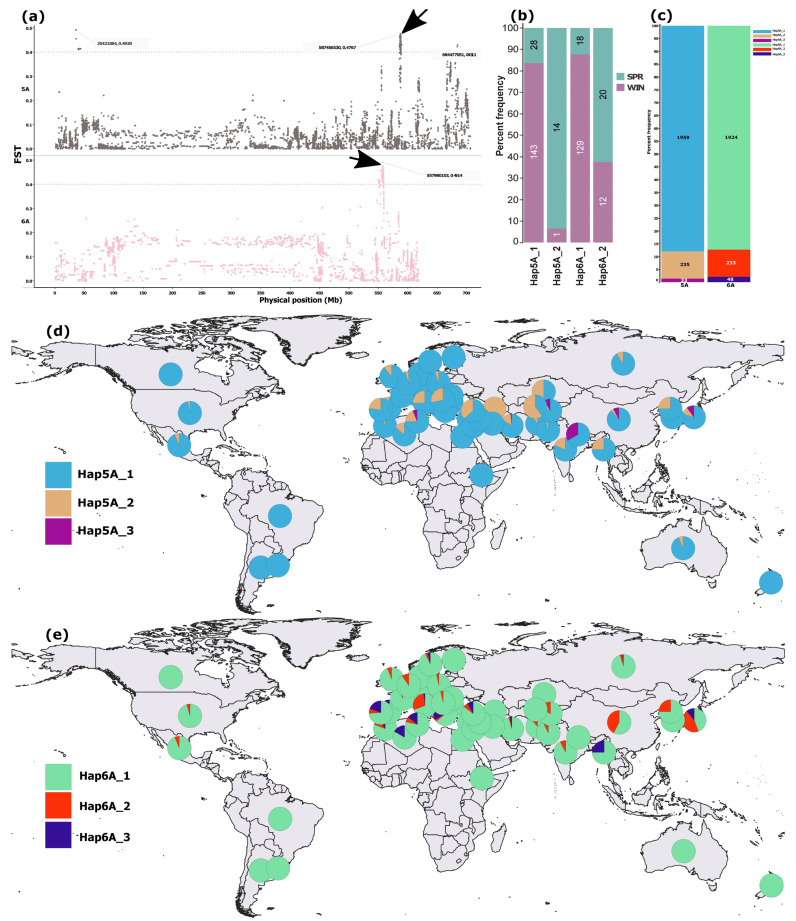
SNP-level *FST* differentiation between spring and winter wheat cultivars and the distribution of haplotypes reconstructed from the top 20 high-*FST* SNPs within differentiated regions on chromosomes 5A and 6A. (**a**) Manhattan plots highlighting differentiated regions on 5A and 6A. (**b**) Frequencies of major haplotypes (defined as those present in ≥10 accessions) reconstructed from the 20 high-*FST* SNPs within the 5A and 6A regions indicated by black arrows, shown for spring and winter cultivars (n = 188). (**c**) Proportional representation of major haplotypes across the full dataset (n = 2240). (**d**,**e**) Geographic distribution of major haplotypes from the 5A and 6A regions, respectively. In (**b**), colors denote growth types, whereas in (**d**,**e**), each color represents a haplotype.

SNP-level *FST* analysis of the full merged dataset (n = 4) detected no clearly differentiated loci between spring and winter types (Figure S1). However, focusing on the cultivar subset uncovered six regions with SNPs showing *FST* > 0.4 on chromosomes 1D, 4B, 5A, and 6A (Figure S2). The strongest signals occurred on 5A and 6A, a single sharp peak at 549–559 Mb on 6A, and two distinct regions on 5A (35–40 Mb and 586–589 Mb) (Figure 3a), the latter coinciding with the sole pronounced peak on 5A from the IPK genome scan (Figure 1) and the most differentiated region from the chromosome 5A scan (Figure 2a).

Haplotype reconstruction from the top 20 SNPs in the 5A (586–589 Mb) and 6A (549–559 Mb) regions revealed two predominant haplotypes in the cultivar subset (n = 188) (Figure 3b) and an additional low-frequency haplotype for each region in the merged dataset (n = 2310), present in ≥10 accessions (Figure 3c). The variation in frequency of the two predominant haplotypes between spring and winter wheat cultivars was more pronounced for 5A than 6A (Figure 3b). The third haplotypes from both regions were rare, occurring in fewer than 3% of accessions in the merged dataset (Figure 3c).

**Figure 4:**
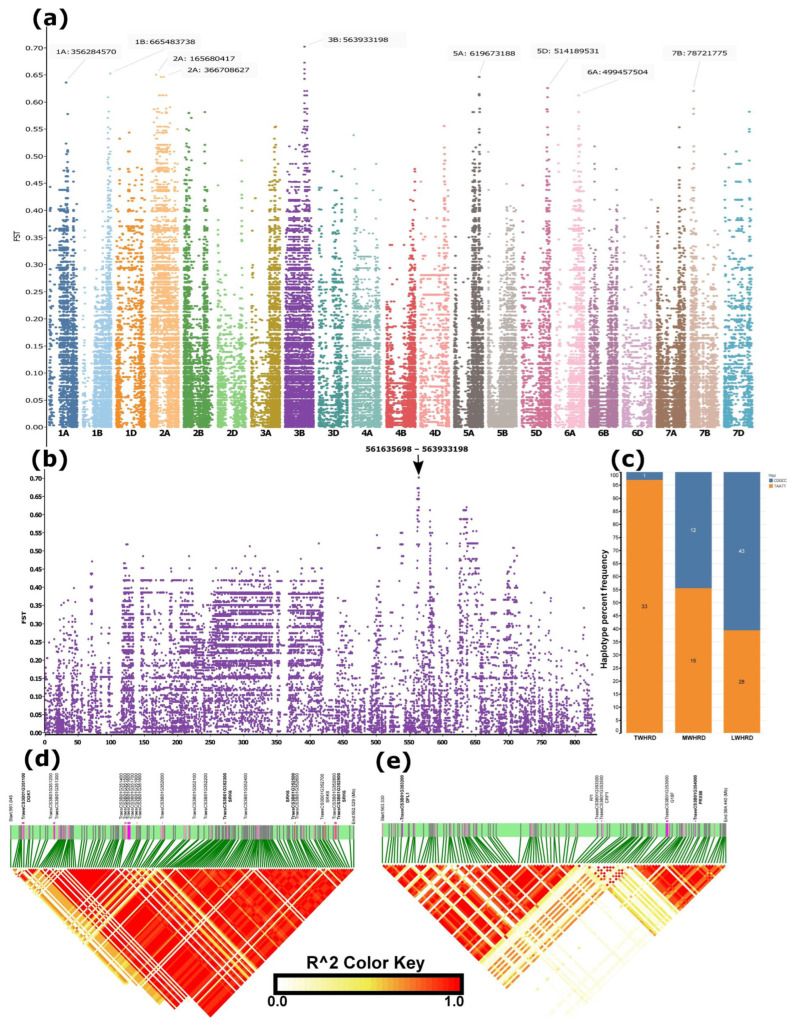
SNP-level *FST* differentiation between winter wheat accessions adapted to minimum temperatures during the coldest quarter (December–February) below –12 °C and above 0 °C, and the genomic regions distinguishing these groups. (**a**) Manhattan plot of genome-wide differentiated regions. (**b**) Enlarged view of chromosome 3B, which harboured the most strongly differentiated region. (**c**) Distribution of haplotypes constructed from the five top high-*FST* SNPs among true winter-hardy (TWHRD, adapted to <–9 °C), medium winter-hardy (MWHRD, adapted to –9 to −5 °C), and low winter-hardy (LWHRD, adapted to >−5 °C) groups. (**d**,**e**) Linkage disequilibrium (LD) blocks within the differentiated region on chromosome 3B. Candidate genes potentially contributing to cold tolerance are indicated above the green bars in bold fonts.

The predominant haplotypes from both 5A and 6A regions were broadly distributed across the global wheat collection (Figure 4d,e). The second most common haplotype on 5A occurred at higher frequencies among West Asian and Mediterranean accessions (Figure 3d), whereas the third most common haplotype on 6A was comparatively enriched in Mediterranean accessions (Figure 3e).

SNP-level *FST* between winter accessions groups adapted to minimum temperature regimes during the coldest quarter (December, January, and February), which was below −12 °C and above 0 °C, extracted from a US–Chinese cultivar-dominated dataset, which included geographic coordinates of accessions for climate data download, detected regions with *FST* > 0.6 on chromosomes 1A, 1B, 2A, 3B, 5A, 5D, 6A, and 7B, with the highest *FST* (*FST* > 0.7) falling in a region on 3B spanning 561 and 564Mb (Figure 4a,b, Table S2). The haplotype based on the five most differentiated SNPs in this region resulted in two haplotypes: ‘CGGCC’ and ‘TAATT’ (Table S3). Haplotype frequency analysis based on a total of 132 (those with climate data) winter accessions grouped as winter-hardy (TWHRD) (<−9 °C, n = 34) (Table 1 and Table S3), moderately hardy (MWHRD) (−9–5 °C, n = 27), and low winter-hardy (LWHRD) (>−5 °C, n = 71) indicated that the first haplotype increased from <3% to over 60% from TWHRD to LWHRD, and appeared as ~45% in MWHRD (Figure 4c). Of the 34 TWHRD, 32 carried a single haplotype constructed based on the top 30 high-*FST* SNPs, and of the remaining two, one appeared to harbour a haplotype that was mismatched with the predominant one by a single allele (out of the 30) at a heterozygous SNP (Table 1 and Table S3). The TWHRD group predominantly carried identical haplotypes, constructed from the top 20 high-*FST* SNPs distinguishing spring and winter wheat types in the merged data, across both strongly differentiated regions on chromosomes 5A and 6A (Figure 3b, Table 1 and Table S3). The sole exception was a Turkish accession (TUR_WINLR1735B), which harboured the alternative haplotype within the differentiated region of chromosome 5A. The TWHRD comprised accessions known for winter hardiness, including the USA benchmark winter-hardy cultivar, Nekota (Table 1 and Table S3).

LD analysis over the entire segment (561 and 564 Mb) of the 3B most differentiated region was divided into two subregions and three LD blocks (Figure 4d,e) The first subregion fell between ~561.5–562.5 Mb in a single LD block (Figure 4d), whereas the second subregion spanned ~563.3 to 564.5 Mb, harbouring two LD blocks (Figure 4e). The top five high *FST* SNPs fell in the second subregion (Table S2).

Information in the first three columns was obtained from Ma et al., 2025 [8]. TMin represents the average minimum temperature during the coldest quarter of the year (December–February). Values of TMin indicate temperature thresholds (e.g., −20 = TMin < −20 °C). 330SNP-3B denotes haplotypes constructed from the 30 most differentiated SNPs between accessions adapted to TMin < −12 °C and >0 °C. Similarly, 20SNP-5A and 20SNP-6A refer to haplotypes constructed from the 20 most differentiated SNPs distinguishing spring and winter wheat cultivars on chromosomes 5A and 6A (Figure 3a), respectively.

**Figure 5:**
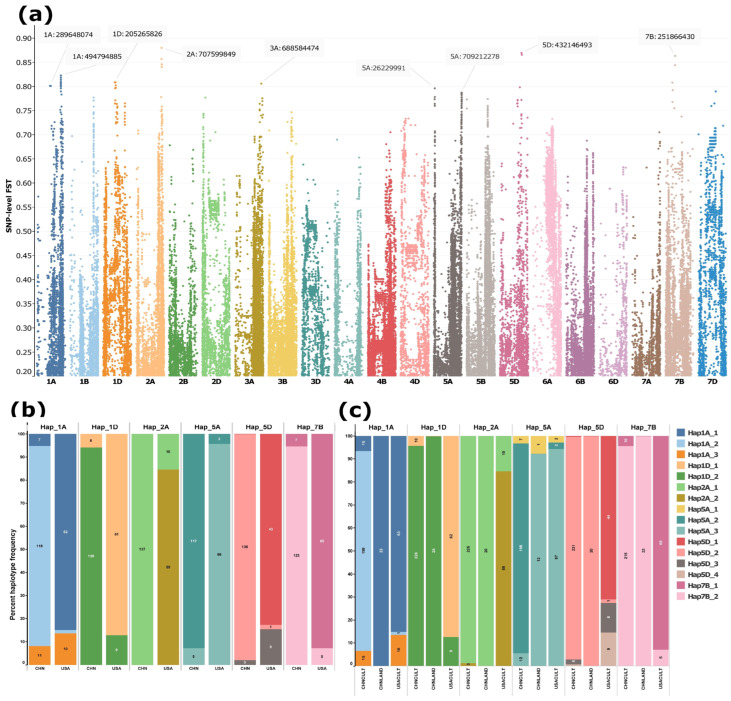
SNP-level *FST* differentiation between Chinese and USA winter wheat cultivars and haplotype frequencies at the six most strongly differentiated loci. (**a**) Manhattan plot of SNP-level *FST* values between Chinese and U.S. winter cultivars. (**b**) Frequencies of major haplotypes (present in ≥10 accessions) at the top differentiated loci in Chinese and USA winter cultivars. (**c**) Distribution of haplotypes across all Chinese (CHNCULT) and USA (USACULT) cultivars as well as Chinese landraces (CHNLAND). Haplotypes were reconstructed from 5 to 30 high-*FST* SNPs (*FST* > 0.75) per locus, depending on the number of SNPs exceeding the threshold. Each color denotes a distinct haplotype. The *y*-axis represents haplotype frequency, with labels on each bar indicating both the percentage and absolute number of cultivars carrying the corresponding haplotype.

SNP-level *FST* analysis between Chinese and U.S. winter wheat cultivars revealed a highly differentiated genomic region on chromosome 2A (spanning ~707.1–715.2 Mb), with several SNPs exhibiting *FST* values approaching 0.9 (Figure 5a; Table S4). Additional differentiated regions were detected on chromosomes 1A, 1D, 5A, 5D, and 7B. Haplotypes constructed from high-*FST* SNPs within these regions showed marked divergence between Chinese and U.S. winter cultivars (Figure 5b). Notably, a considerable fraction of U.S. cultivars also carried the haplotype predominant in Chinese cultivars, but this was not true for the other way around (Figure 5b).

When the analysis was extended to the full dataset, including Chinese cultivars (both winter and spring types), Chinese landraces, and U.S. cultivars, distinct patterns emerged. In the differentiated regions on 1A and 5A, Chinese landraces predominantly carried haplotypes common in U.S. cultivars, HAP1A_1 and HAP5A_3, whereas modern Chinese cultivars carried the alternative haplotypes HAP1A_2 and HAP5A_2 (Figure 5c). For the remaining detected regions on other chromosomes (1D, 2A, 5D, and 7B), HAP1D_2, HAP2A_1, HAP5D_2, and HAP7B_2 dominated in both the cultivars and landraces of the Chinese lines. The Chinese landraces carried a single haplotype (complete fixation) across all targeted genomic regions, with the exception of the differentiated region on 5A (Figure 5c). HAP2A_1 on 2A was nearly fixed in Chinese cultivars (225 of 228 accessions) (Figure 5c) and represented the sole haplotype in Chinese winter cultivars (Figure 5b). The U.S. cultivars harboured two unique/private haplotypes on 5D (Figure 5c). No private haplotypes were observed in either Chinese cultivars or landraces.

### 2.2. Wheat Production Under Extreme Winter Temperatures in Temperate Zones

**Figure 6:**
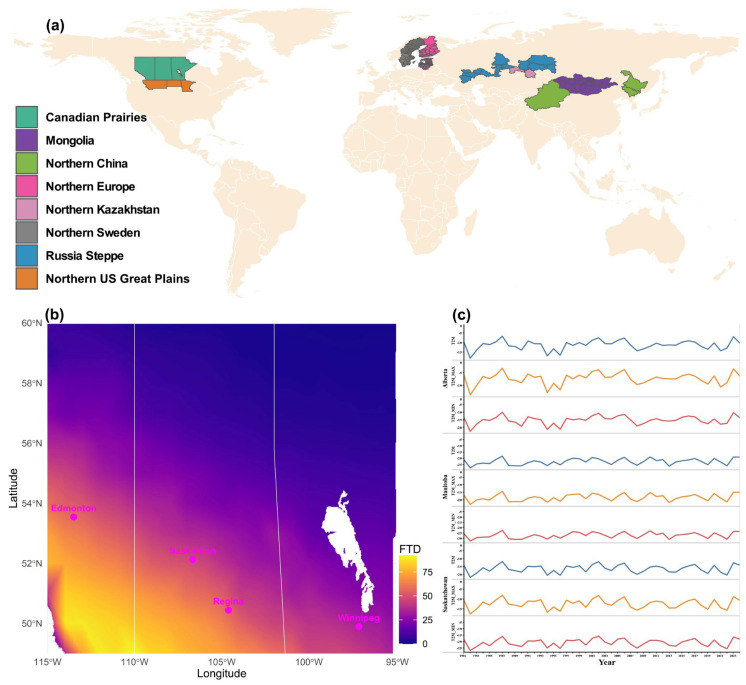
Wheat-growing regions in temperate zones predominantly produce spring wheat, with freeze–thaw frequency in the Canadian Prairies as a representative example. (**a**) Regions where extreme winter cold restricts winter wheat cultivation, resulting in predominant spring wheat production. (**b**) Freeze–thaw events from November to March (1981–2024), derived from NASA POWER minimum and maximum temperature data for the major wheat-producing Prairie provinces in Canada. Bars indicate the number of freeze–thaw days (FTD) within the specified period. Water bodies, including Lake Winnipeg, are shown in white; provincial borders are delineated by white lines. Representative cities are highlighted in pink. (**c**) Average temperature records of the Prairie provinces during the coldest quarter of the year (December–February) from 1981 to 2024; T2M = daily average temperature at 2 m, T2M_Max = maximum temperature at 2 M, T2M_Min = minimum temperature at 2 m.

Multiple areas in the temperate region, accounting for over 15% of the global wheat-growing regions, are still reliant on spring wheat due to a lack of winter wheat cultivars that consistently survive their extremely cold winter temperatures (Figure 6a). Not only are extreme, low winter temperatures an issue, but wheat also encounters frequent mid-winter freeze–thaw cycles and limited snow cover to serve as insulation to protect the crown of wheat seedlings, which are critical challenges for winter wheat survival.

The Canadian Prairie provinces (Alberta, Saskatchewan, and Manitoba) are among the major wheat-producing areas of the world, with major spring wheat production, as the success of winter wheat production is heavily hampered by both extremely cold winters and intermittent mid-winter freeze–thaw cycles. However, freeze-thaw frequencies significantly vary across the provinces (Figure 6b). The eastern, northeastern, and northern regions of the Canadian Prairies, which together comprise the majority of the Prairies’ landscape, experienced zero or near-zero winter thaw days between November and March. By contrast, the central, southwestern, and southern sectors of the Prairies exhibited a markedly higher frequency of thaw events. The greatest incidence of freeze–thaw days, as high as ~90 days, occurred in the southernmost areas, particularly within the latitudinal range of ~49–50° N and longitudinal range of ~109–114° W, corresponding to the southern border regions of Saskatchewan and Alberta (Figure 6b). Major urban centres, including Edmonton, Regina, Saskatoon, and Winnipeg, are situated within a transitional belt characterized by medium-to-high thaw frequency. Among these, Edmonton and Regina recorded approximately 50 thaw days, while Winnipeg, with slightly over 25 days, experienced the lowest frequency within this urban belt (Figure 6b). The pattern of average daily, minimum, and maximum winter temperature records (December to February) appeared similar for all three provinces, where the lowest and the highest were recorded in 1982 and 1987, respectively (Figure 6c).

## 3. Discussion

The substantial gap between the global wheat yield record (~18 t/ha) and the average yield in most major wheat-producing countries (~3.5 t/ha) underscores the need for further intensification through advanced breeding strategies and other practices. The superior yields observed in regions dominated by winter wheat highlight the intrinsic yield advantage of the winter wheat type, suggesting potential benefits to expanding winter wheat cultivation into temperate areas currently dedicated to spring wheat.

The growing wealth of genomic and environmental data now allows for the identification of variants carrying alleles/haplotypes associated with key traits, including cold, drought, and stress tolerance. These insights not only facilitate the development of high-yielding, resilient cultivars but also enable precise adaptation of germplasm to specific environments, introducing the concept of cultivar–niche matching for optimized production. The results of the present study provide valuable information on genomic regions associated with cold tolerance and identify accessions harbouring favorable alleles, serving as potential resources for winter wheat breeding programs.

In the present study, the genomic regions identified through genome scanning and SNP-level FST are consistent with previous studies that highlighted major vernalization and cold-resistant loci. The multiple signature regions on 5A between ~526 and 703 Mb coincide with loci harbouring key vernalization and frost tolerance loci on this chromosome. The region spanning ~526–544 Mb overlaps the Fr-2 locus, which contains clusters of CBF transcription factors that act as central regulators of the cold response by activating a broad network of cold-inducible genes conferring freezing tolerance in wheat [9]. The allelic and haplotype diversity observed within this interval underscores the adaptive potential of accessions to contrasting temperature regimes.

The conspicuously differentiated interval spanning 586–589 Mb on chromosome 5A (Figure 2 and Figure 3a) represents a major target of selection. This region encompasses the *VRN-A1* gene together with the *Fr-1* locus [10]. However, it remains unclear whether *VRN1* and *Fr-1* correspond to two distinct genetic entities or the observed effects are attributable to a pleiotropic role of *VRN1*, functioning both as a vernalization determinant and as a contributor to freezing tolerance. *VRN1* plays a pivotal role in integrating developmental timing with stress adaptation by linking the vernalization requirement to cold tolerance, thereby ensuring synchronization of flowering with winter survival capacity [11]. The recurrence of this region across independent datasets highlights its consistent relevance as a target for wheat improvement through selection [4]. Notably, allelic variation at this locus frequently differentiates wheat accessions into spring and winter types, reflecting its central role in both vernalization and cold acclimation pathways [4,11,12].

The region is well known for harbouring the *MADS-box* genes *TRAESCS5A02G391700* (*VRN1*) and *TRAESCS5A02G391800* (*MADS34*), respectively [10]. *TRAESCS5A02G391700* was characterized as *APETALA1* (*AP1/WAP1*), the key vernalization gene, VRN1, whereas *TRAESCS5A02G391800* (*MADS34*) appears to be a homolog of *AGAMOUS-Like-Globosa-like 1* (*AGLG1*), the flowering gene operating in the downstream vernalization pathway [13,14]. Distal and downstream from *MADS34, TRAESCS5A02G391900* is consistent with *cytochrome B5* (*CYB5*) [13]. This gene is involved in biological processes conferring multiple stress responses [15,16]. We could not find any wet-lab result evidence; however, databases such as KnetMiner list *TRAESCS5A02G391900* (*B5#6*) as one of the genes that are involved in tolerance to extreme climate conditions, such as minimum temperature and other factors, during the coldest quarter of the year (Table 2).

The precise physical overlap of the 5A 586–588 Mb interval with one of the two regions exhibiting the strongest differentiation between spring and winter wheat types (Figure 3a) further underscores the functional importance of this locus in mediating adaptation to contrasting environmental conditions. This convergence of differentiation signals suggests that allelic variation within this region has likely been subject to strong selective pressures associated with vernalization requirement and cold tolerance, enabling wheat to optimize growth and reproductive timing under distinct climatic regimes.

The distal interval on chromosome 5A (~698–703 Mb) harbours two *VRN2* (*ZCCT*) paralogs and *VRN3*, the wheat homolog of the Arabidopsis *FLOWERING LOCUS T* (*FT*) gene [51]. Together, these loci modulate the balance between flowering induction and prolonged cold adaptation (Chen & Dubcovsky, 2012) [52]. Functional interactions among these regulators are well-established: *VRN1* and *VRN3* act synergistically, with the upregulation of one enhancing expression of the other while repressing *VRN2*, and vice versa [53,54]. Interestingly, upregulation of *VRN2* has been associated with enhanced cold tolerance, while it leads to loss of function at *Fr-1*, which colocalizes with *VRN1* [54].

Genomic regions identified through SNP-level FST analyses between accessions adapted to extreme minimum winter temperatures (<−12 °C) and those from milder climates (>0 °C) are likely to harbour genes contributing to cold tolerance. Notably, a differentiated region on chromosome 1B, spanning approximately 665.0–665.9 Mb, contains a higher density of genes implicated in cold response and tolerance, underscoring its potential importance in adaptation to low-temperature environments. Within this interval, the *PIN3* gene, encoding an auxin efflux carrier protein, has been shown to respond to multiple abiotic stresses, including low temperatures, potentially disrupting auxin transport under chilling conditions [55]. The region also harbours a gene encoding mitogen-activated protein kinase (MAPK), a signalling component widely implicated in plant responses and adaptations to various abiotic stresses, including cold stress [56,57,58]. Specifically, the *MPK17* gene ortholog within this region has been reported to respond to cold stress in both maize and Arabidopsis [56]. The other notable candidate in this region, FT1 (*VRN3*) [51], is a well-characterized integrator of the vernalization pathway in wheat; prolonged cold exposure relieves repression of *FT1*, enabling its induction and the promotion of flowering [51,59]. Additionally, *Chloroplast Unusual Positioning 1* (*CHUP1*), also present in this region, influences chloroplast positioning, photosynthetic efficiency, and carbohydrate transport, processes that can be critical under cold–light-stress conditions [60,61].

The *diacylglycerol kinase* (*DGK1*) ortholog gene, *TRAESCS3B02G351100*, in the region on 3B with the highest FST between the extreme and mild cold-temperature-adapted lines (Figure 4), is one of the key candidate genes associated with extreme cold tolerance. This gene is known to respond to low temperatures in Arabidopsis [62]. The gene is involved in biosynthesis of lipid messengers in response to multiple abiotic stresses [63,64]. In wheat, *TaDGKs* have been upregulated under salt and drought conditions [65].

The gene encoding peroxidase 56 (PRX56, *TRAESCS3B02G354000*), located within the second subregion’s second LD block of the differentiated region on chromosome 3B (Figure 4e), represents another strong candidate associated with cold tolerance. Class III peroxidases such as PRX56 are key components of the antioxidant defense system, functioning in the detoxification of reactive oxygen species (ROS) and in cell wall remodelling under stress. Consistent with this role, overexpression of *PRX* genes has been shown to enhance cold tolerance in several plant species [66]. In wheat, transcriptomic studies have reported the upregulation of *TaPRX* genes in response to low-temperature stress, contributing to improved tolerance not only to cold but also to a broader spectrum of biotic and abiotic stresses [67].

The *Dwarf and Low-Tillering 1* (*DFL1*) gene (*TRAESCS3B02G353200*), located within the first LD block of the second subregion that also harbours the SNPs with the highest FST values, emerges as another candidate potentially associated with cold tolerance. DFL1 encodes an auxin-responsive Gretchen Hagen3 (GH3) family protein functioning as an IAA-amido synthetase, which reduces auxin signalling through the conjugation of free indole-3-acetic acid (IAA) to amino acids [68]. By modulating auxin homeostasis, *DFL1* influences a wide spectrum of developmental and stress-related traits. In wheat, *TaGH3* genes are expressed in multiple abiotic stresses, including osmotic stresses, which can also be associated with cold-induced dehydration [69]. According to the KnetMiner database, the *DFL1* in this subregion is involved in modulation of several traits, including spike architecture (SALTTL), auxin content, cold, drought and heat tolerance, male sterility, heterosis, root length, and plant height. Although its role in cold tolerance is likely indirect, *DFL1* may contribute by reprogramming auxin-mediated growth–stress trade-offs, restricting growth, and reallocating metabolic resources toward defense pathways during exposure to low-temperature stress.

Given that this genomic region on chromosome 3B exhibited the strongest differentiation between accessions adapted to extremely low versus mild winter minimum temperature regimes, together with the presence of biologically plausible candidate genes and pronounced haplotype frequency variation among the TWHRD, MWHRD, and LWHRD groups (Figure 4e), it invites further detailed investigation. These patterns strongly suggest that the region plays a pivotal role in conferring tolerance to environments characterized by extremely cold winters. Notably, however, the region is subdivided into distinct LD blocks, and the leading candidate genes *DGK1* and *PRX56*, based on previously described biological function, are not in strong linkage disequilibrium with the most highly differentiated SNPs. This discrepancy underscores the need for fine-scale mapping and functional validation to resolve the genetic architecture of this genomic region and to assess additional candidate genes that may underlie extreme cold tolerance.

The observed divergence between Chinese and U.S. winter wheat cultivars at specific genomic regions likely reflects distinct selection targets shaped by the breeding histories of the two countries, encompassing agronomic performance as well as resistance to abiotic and biotic stresses [8,70]. The occurrence of the same nearly fixed major haplotype in both Chinese cultivars and landraces across differentiated regions on chromosomes 1D, 2A, 5D, and 7B, in contrast to the distinct haplotypes observed in U.S. cultivars at the corresponding regions (Figure 5c), indicates that selection has been shaped in a country-specific manner, extending beyond recent breeding programs to historical landrace development. Notably, the highly fixed haplotype in Chinese landraces and cultivars within the differentiated region on chromosome 2A (spanning ~707.1–715.2 Mb) coincides with the *Yr86* locus, which confers stripe rust resistance and has long been recognized as a major target in Chinese wheat breeding [71,72]. In contrast, no well-characterized selection target has been documented for U.S. cultivars within this genomic interval. This region harbours a haplotype that is carried by ~85% of US lines, while it is present in only ~1% (3 out of 248) of Chinese lines (Figure 6b,c), suggesting the presence of potential, as yet uncharacterized, targets of selection for US breeding programs.

The major haplotypes in Chinese landrace and US cultivars are similar, although the Chinese cultivars harbour different major haplotypes in differentiated regions on 1A (494.5–495.1 Mb) and on 5A (703.1–709.2 Mb), aligning with the performance of Chinese cultivars [73] partly reflecting the predominant cultivar types between the two countries [8]. However, the total difference is a function of combined factors, including more intensive input use and double-cropping systems in China (higher fertilizer and irrigation intensity), a sustained breeding emphasis on yield and disease resistance, and measurable genetic gains from modern Chinese breeding programs [73,74].

The occurrence of haplotypes common to Chinese germplasm within a subset of U.S. cultivars likely reflects historical introductions of Chinese material into U.S. breeding programs, driven by the recognized superior performance of Chinese cultivars and/or breeding lines. For example, Xiaoyan 6 has contributed to over 80 released cultivars in China and served as a donor of disease resistance, drought tolerance, and high yield [75], with alien chromatin from wheatgrass conferring critical adaptive traits, including cold tolerance. Similarly, spring wheat Sumai 3 has been widely utilized worldwide as a source of Fusarium head blight (FHB) resistance. However, despite the well-documented performance of Chinese cultivars under multiple biotic and abiotic stresses, including winter hardiness, we could not trace reports of Chinese germplasm contributing to breeding programs outside China, with the exception of Sumai 3. However, the absence of formal documentation does not preclude the possibility that some programs have incorporated Chinese germplasm, either directly or indirectly, as unreported parental lines.

Winter hardiness in wheat is a complex, polygenic trait shaped by multiple loci distributed throughout the genome. It integrates several physiological and molecular processes, including cold acclimation, vernalization, and stress tolerance, all of which are indispensable for survival and productivity in low-temperature environments. Central to this regulatory network are the *Fr* (*Frost resistance*) loci, which govern the expression of *C-repeat Binding Factor* (*CBF*) *transcription factors*. The *CBF* genes activate downstream cold-responsive pathways, including the Wheat *Cold-Regulated* (*WCOR*) genes, which encode structural and protective proteins that stabilize cellular functions during freezing stress. These pathways are further modulated upstream by the *VRN1* (*Vernalization1*) gene, a major determinant of flowering time [11]. Notably, the same low-temperature cue elicits contrasting regulatory outcomes depending on developmental stage and season: a low-temperature threshold that suppresses *VRN1* expression, thereby permitting upregulation of *CBF* and *WCOR* to establish cold acclimation in autumn, and increases VRN1 transcription level, which promotes flowering and concurrently downregulates *CBF* and *WCOR* in spring [11,54].

The dual role of temperature underscores the dynamic and finely tuned nature of wheat’s adaptive response, in which identical thermal cues are differentially interpreted through the interaction of season-specific environmental conditions and the plant’s developmental stage to optimize both survival and reproductive success. The contrasting responses of *VRN1* to the same low-temperature threshold are, at least in part, modulated by its sensitivity to photoperiod and the prevailing temperature regimes. In autumn, short days and progressively declining temperatures favor the downregulation of *VRN1*, thereby promoting the induction of the *CBF* and *WCOR* genes to initiate cold acclimation. In the spring, the same low-temperature threshold occurs under longer day lengths and rising temperatures, conditions that favor the upregulation of *VRN1* to promote floral initiation while downregulating cold-responsive pathways [11].

The switch between vernalization and *CBF*-mediated cold acclimation represents a finely tuned process, triggered at thermal thresholds generally above freezing. This timing allows plants to accumulate protective *CBF* transcripts and downstream metabolites, enhancing their resilience during the winter. The duration of this acclimation phase is directly correlated with hardiness; longer acclimation typically confers greater survival capacity [54]. However, even the most winter-hardy wheat cultivars exhibit a limit in their tolerance. *VRN–CBF*-mediated tolerance, while central, does not provide a complete explanation of winter survival, suggesting additional layers of adaptations [76].

The 34 accessions identified in this study as tolerant to extreme minimum winter temperatures (<−9 °C; Table 2) represent highly promising pre-breeding candidates for cultivation in regions subject to severe winter conditions, including the Canadian Prairies, the Siberian Steppe of Russia, northern Kazakhstan, northern Europe (including the Baltic states), and the North China–Mongolia wheat belt (Figure 6). Their potential is further underscored by the inclusion of top US winter-hardy varieties, such as Nekota, Harding, and Ankor, which are adapted to the Northern Great Plains and the Rocky Ridge region of Colorado, highlighting the relevance of all 34 accessions for extremely cold winter wheat breeding.

The predominance of identical haplotypes in the differentiated region of chromosome 3B, together with the 5A and 6A regions that distinguish winter and spring wheat, indicates that these accessions carry alleles commonly associated with established winter wheat types. The fact that only a single accession from China and another from Turkey displayed alternative haplotypes in the 3B and 5A differentiated region, respectively, highlights the overall stability of the predominant haplotype across diverse geographic origins. This genetic consistency not only reinforces the potential of these accessions for breeding programs targeting extreme cold environments but also suggests that these haplotype loci may have been subject to strong selective pressures in harsh winter climates, preserving alleles essential for survival. Such conservation further hints at the value of these accessions as pre-breeding material for winter wheat breeding programs in regions experiencing severe frost and prolonged low temperatures.

## 4. Materials and Methods

### 4.1. Data

Three genotype datasets generated through whole-genome resequencing were used in this study: (1) a predominantly winter wheat panel from Europe (n = 768), genotyped with approximately 215 million SNPs [77]; (2) the spring wheat-dominated Watkins collection (n ≈ 1051), genotyped with approximately 260 million SNPs [78]; and (3) a panel primarily composed of Chinese and U.S. cultivars along with other landraces (n = 491) [8]. These datasets were retrieved from the ENA Browser, Browse—BioProject—CNCB-NGDC, and Genome Variation Map (accession: GVM000359), respectively. Herein, the three datasets are denoted as WGIPK (from the German Leibniz-Institut für Pflanzengenetik und Kulturpflanzenforschung, IPK), WGWat (Watkins collection), and WGCHNUS (Chinese and U.S. lines). Each dataset was processed individually, applying quality control filters to retain variants with a maximum of 3% missing genotype calls and a minor allele frequency (MAF) of at least 5%. The datasets were also merged and further filtered to include variants with no more than 3% missing data and a MAF threshold of 5% in the climate data, including precipitation, relative humidity, maximum and minimum, daily average air temperature (at 2 M), and daily average land surface temperature were obtained from the NASA POWER agroclimatology platform (https://power.larc.nasa.gov/data-access-viewer/, (accessed on 8 October 2025). These data were extracted for 220 accessions in the WGCHNUS dataset, using the geographic coordinates provided in the passport data of each accession. Although coordinate information was also available for other datasets such as WGWat, in most cases, these coordinates reflected only the country of origin rather than the specific collection sites of the samples. Consequently, we excluded those coordinates from climate data extraction to maintain spatial accuracy and avoid potential misrepresentation.

### 4.2. Analyses

#### 4.2.1. Patterns of Adaptive Genetic Signatures

To capture the major genetic regions across the genome reflecting selection and adaptive signatures, principal component-based genome scans were performed on quality-controlled final individuals of the merged dataset using pcadapt v4.4.1 in R [79]. These scans were performed both genome-wide and chromosome wide. Similar pcadapat analysis was performed on the IPK datasets (WGIPK). The selection of the datasets was based on the representation of landraces and winter wheat types. The results were visualized as Manhattan plots using Tableau Public v2025.2 (https://public.tableau.com, accessed on 16 July 2025).

#### 4.2.2. SNP-Level FST Analyses of Growth Habit and Winter Cold Adaptation

To trace major genomic regions differentiated between spring and winter wheat types, subsets of accessions representing these two growth habits were extracted from the merged and WGCHNUS datasets, both of which contain relatively balanced numbers of spring and winter wheats. For each selected data subset, SNP-level FST analysis was then performed to detect highly differentiated genomic regions between the spring and winter types. Spatial distribution of major haplotypes (present in ≥10 accessions) constructed based on SNPs from selected most differentiated genomic regions were visualized using Quantum Geographic Information System (QGIS) v3.24 (https://qgis.org, accessed on 16 July 2025).

Assuming that variation exists among winter wheat types adapted to both extreme and mild winter environments, we further conducted an FST analysis between winter wheat accessions originating from environments characterized by average minimum temperatures of the coldest months (December to February) below –12 °C and above 0 °C, using the winter subsets of the WGCHNUS dataset. While this analysis focused on accessions adapted to extreme temperature conditions, the entire winter subset was additionally stratified into three groups based on their degree of winter hardiness: true winter-hardy (TWHRD; <–9 °C), medium winter-hardy (MWHRD; –9 to –5 °C), and low winter-hardy (LWHRD; >–5 °C). The winter period temperature data were obtained from downloaded environmental data based on the coordinates of the accessions.

To elucidate patterns of genomic differentiation between winter wheat breeding programs in China and the United States, we also performed an SNP-level FST analysis to identify genomic regions exhibiting the greatest divergence between cultivars from the two countries using winter wheat cultivars extracted from the WGCHNUS dataset. For each of the top differentiated regions, haplotypes were constructed using the top 30 high FST SNPs within the interval, and haplotype frequencies were compared across Chinese and U.S. cultivars. Genes located within the top five highly differentiated regions were retrieved from KnetMiner (https://app.knetminer.com/plants-lite/Triticum_aestivum, (accessed on 16 July 2025)), along with their annotated functional roles, and further examined to assess their potential relevance as region-specific breeding targets. To identify the genes in linkage disequilibrium (LD) with the differentiated SNPs, we conducted LD analysis and visualized LD block structures using LDBlockShow v1.41 [80], where the genes were annotated within the LD block visualizations.

#### 4.2.3. Freeze–Thaw Risk Mapping to Inform Niche-Specific Winter Wheat Breeding

In regions subject to intermittent freeze–thaw cycles, cold hardiness alone is insufficient to guide effective winter wheat breeding. Therefore, it is crucial to account for specific niche conditions when designing breeding and agronomic strategies. To this end, we estimated the number of freeze–thaw days across the Canadian Prairie provinces, spanning approximately 49–60° N latitude and 95–120° W longitude. The Canadian Prairies were targeted because they represent one of the largest wheat-growing regions globally and are characterized by both extreme cold and localized freeze–thaw events. By quantifying freeze–thaw frequency across this region, we can delineate areas of high and low winter-thaw risk, thereby informing niche-specific breeding and management approaches. Freeze–thaw days were calculated using daily minimum and maximum temperature records from November to March for the years 1981–2024, obtained from NASA POWER. A freeze–thaw day was defined as any day with a minimum temperature (Tmin) ≤ 0 °C and a maximum temperature (Tmax) ≥ 0 °C. Spatial analyses and mapping were performed in R (ver. 0.6-1) using package sf [81].

## 5. Conclusions

The findings of this study provide new insights into adaptation to extreme cold winters in wheat, revealing multiple genomic regions, in addition to the well-established loci previously reported. These results further substantiate the complex, polygenic nature of cold tolerance, which is governed by intricate interactions among gene networks, physiological processes, and environmental factors. This complexity underscores the need for a holistic breeding strategy that integrates genetic improvement with agronomic innovation, niche-specific environmental optimization, and the strategic deployment of adaptive germplasm. Such optimization should also consider the timing of planting to minimize exposure to early freeze–thaw cycles, which often pose critical risks at the onset of true winter.

Given that wheat is inherently adapted to vernalization and overwintering, the development of cultivars with enhanced extreme cold tolerance is both a realistic and attainable objective, provided that it is supported by focused research, sustained investment, and technological innovation. Historical agricultural transformations, such as the incorporation of dwarfing genes that fueled the Green Revolution and research successes in other crops, demonstrate that persistent innovation can overcome formidable environmental constraints. Encouragingly, recent breeding efforts have already produced lines exhibiting winter survival rates comparable to, or approaching, that of the benchmark winter-hardy cultivar Norstar. Hence, continued intensive research aimed at enhancing extreme cold tolerance, improving resilience to freeze–thaw cycles, and optimizing complementary traits such as disease resistance and yield holds promise for the development of reliably adapted cultivars capable of thriving in the most severe winter environments in current wheat-growing regions and beyond.

Future breeding programs should therefore combine genomic selection with precise and dynamic phenotyping approaches, supported by high-throughput tools such as drone-based imaging, environmental sensing, and predictive modelling. The increasing accessibility of multidimensional datasets, including genomic, environmental, and long-term phenotypic records, together with guiding databases and advances in computational analytics, including artificial intelligence, provides a foundation for informed breeding decisions. These integrated resources position extreme cold adaptation in winter wheat as a key frontier in modern crop science, with transformative implications for global wheat production.

## Figures and Tables

**Table 1:** Accessions adapted to regions experiencing <−9 °C and the haplotypes they carry at 3B, 5A, and 6A differentiated regions.

Sample ID	Name	Area Adapted	TMin (°C)	30SNP_3B	20SNP_5A	20SNP_6A
1537B	TJK03-194	Tajikistan, Amir ridge	−20	HAP1	5A_HAP1	6A_HAP1
1577B	TJK03-195	Tajikistan, Amir ridge	−20	HAP1	5A_HAP1	6A_HAP1
1785B	K2003-8	Kazakhstan, Tien-Shan ridge	−15	HAP1	5A_HAP1	6A_HAP1
1541B	WB411W	USA, Montana	−12	HAP1	5A_HAP1	6A_HAP1
1637B	Shavano	USA, Montana	−12	HAP1	5A_HAP1	6A_HAP1
S0059	Early Premium	USA, Montana	−12	HAP1	5A_HAP1	6A_HAP1
S0130	Triumph	USA, Montana	−12	HAP1	5A_HAP1	6A_HAP1
1735B	79TK109-595	Turkey, Erzurum	−12	HAP1	5A_HAP2	6A_HAP1
1761B	TJK2006:298	Tajikistan, Panjakent	−12	HAP1	5A_HAP1	6A_HAP1
1526B	Laredo	USA, Colorado, Rocky ridge	−12	HAP1	5A_HAP1	6A_HAP1
1527B	Bond CL	USA, Colorado, Rocky ridge	−12	HAP1	5A_HAP1	6A_HAP1
1567B	Halt	USA, Colorado, Rocky ridge	−12	HAP1	5A_HAP1	6A_HAP1
1599B	Ripper	USA, Colorado, Rocky ridge	−12	HAP1	5A_HAP1	6A_HAP1
1612B	Thunderbolt	USA, Colorado, Rocky ridge	−12	HAP1	5A_HAP1	6A_HAP1
1626B	Ogallala	USA, Colorado, Rocky ridge	−12	HAP1	5A_HAP1	6A_HAP1
1646B	Oro Blanco	USA, Colorado, Rocky ridge	−12	HAP1	5A_HAP1	6A_HAP1
1664B	Ponderosa	USA, Colorado, Rocky ridge	−12	HAP1	5A_HAP1	6A_HAP1
1670B	Hatcher	USA, Colorado, Rocky ridge	−12	HAP1	5A_HAP1	6A_HAP1
1682B	Above	USA, Colorado, Rocky ridge	−12	HAP1	5A_HAP1	6A_HAP1
1713B	Longhorn	USA, Colorado, Rocky ridge	−12	HAP1	5A_HAP1	6A_HAP1
1714B	Jules	USA, Colorado, Rocky ridge	−12	HAP1	5A_HAP1	6A_HAP1
1723B	Yuma	USA, Colorado, Rocky ridge	−12	HAP1	5A_HAP1	6A_HAP1
1797B	Cisco	USA, Colorado, Rocky ridge	−12	HAP1	5A_HAP1	6A_HAP1
1816B	Prowers	USA, Colorado, Rocky ridge	−12	HAP1	5A_HAP1	6A_HAP1
1835B	Hondo	USA, Colorado, Rocky ridge	−12	HAP1	5A_HAP1	6A_HAP1
1862B	Ankor	USA, Colorado, Rocky ridge	−12	HAP2	5A_HAP1	6A_HAP1
1653B	Tomahauk	USA, Northern Plaines	−9	HAP1	5A_HAP1	6A_HAP1
S0061	XinDong 26	China, Xinjiang Uyghu	−9	HAP3	5A_HAP1	6A_HAP1
1529B	Crimson	USA, Northern Plaines	−9	HAP1	5A_HAP1	6A_HAP1
1582B	Alice	USA, Northern Plaines	−9	HAP1	5A_HAP1	6A_HAP1
1651B	Harding	USA, Northern Plaines	−9	HAP1	5A_HAP1	6A_HAP1
1659B	Nekota	USA, Northern Plaines	−9	HAP1	5A_HAP1	6A_HAP1
1721B	SD97538	USA, Northern Plaines	−9	HAP1	5A_HAP1	6A_HAP1
1755B	Tandem	USA, Northern Plaines	−9	HAP1	5A_HAP1	6A_HAP1

**Table 2:** Genes linked to climate conditions of the coldest quarter of the year at selected high-*FST* loci between spring and winter wheat cultivars.

Gene Accession ID	Gene Name	Position	Climate Link	Reference
*TRAESCS2A02G272400*	*NPL1*	2A:445752512	clim-tmin1, clim-tmin2	[17,18,19]
*TRAESCS2A02G277700*	*QOR*	2A:459926690	clim-tmin12	[20,21]
*TRAESCS2A02G277900*	*QOR*	2A:460899578	clim-tmin12	
*TRAESCS2A02G281200*	*FRU/FIT*	2A:469810800	clim-bio6, clim-bio11, clim-tmax1, clim-tmin1, clim-tmin12, clim-tmin2	[22,23]
*TRAESCS2B02G127700*	*IBR3*	2B:95759390	clim-bio6, clim-bio11, clim-tmin1, clim-tmin2	[21,24,25]
*TRAESCS2B02G127900*	*RPL18*	2B:95797166	clim-bio6, clim-tmin1, tmin2	[26,27]
TRAESCS3A02G149000 *	*CRF1*	3A:132581440	clim-tmin12, clim-tmin2	[28,29,30]
*TRAESCS3A02G150200*	*BASS4*	3A:136567573	clim-bio6, clim-tmin1, clim-tmin12, clim-tmin2	[31,32]
TRAESCS4A02G098600 *	*ERD2*	4A:110636805	clim-bio6, clim-tmin1, clim-tmin2	[33,34]
*TRAESCS4A02G437200*	*EDR2*	4A:707040097	clim-tmin1	[35]
*TRAESCS4B02G274900*	*SbtS*	4B:552829741	clim-bio6, clim-tmax12, clim-tmin1	
*TRAESCS5A02G391900*	*CYB5*	5A:588371644	clim-bio6, clim-tmin1, clim-tmin2	[15,16,36]
TRAESCS5B02G029300 *	*SIS7/NCED3*	5B:29337289	clim-bio11	[37,38,39]
*TRAESCS5D02G360800*	*NAC45*	5D:440369907	clim-tmin12	[40,41]
*TRAESCS6D02G401600*	*RR3*	6D:470632933	clim-tmin12, clim-tmin2	[24,42,43]
*TRAESCS7A02G257200*	*RIN4*	7A:246599963	clim-bio6, clim-tmin1	[44,45]
*TRAESCS7A02G260600*	*AGL9*	7A:253427732	clim-bio6, clim-tmin1, clim-tmin12, clim-tmin2	[46]
*TRAESCS7A02G262600*	*LF/LFR*	7A:258823949	clim-tmin1	[47]
*TRAESCS7B02G302500*	*NSE4A*	7B:539233210	clim-bio6, clim-min1, clim-tmin12, clim-tmin2	[48,49]
*TRAESCS7B02G304700*	*OGR1*	7B:546011345	clim-bio6, clim-tmin1, clim-tmin2	[50]

Clim-tmin1, clim-tmin2, clim-tmin12 = minimum temperature for January, February, and December; clim-bio6 and clim-bio11 = minimum temperature of the coldest month and quarter, respectively. * Genes directly involved in cold tolerance. The data was extracted from the KnetMiner database.

## Data Availability

The original contributions presented in this study are included in the article/Supplementary Materials. Further inquiries can be directed to the corresponding authors.

## Supplementary Materials

The following supporting information can be downloaded at https://www.mdpi.com/article/10.3390/ijms27031568/s1.

## References

[1] UN, FAOSTAT (2023). FAOSTAT Crops and Livestock Products Data.

[2] Shiferaw B., Smale M., Braun H.J., Duveiller E., Reynolds M., Muricho G. (2013). Crops That Feed the World 10. Past Successes and Future Challenges to the Role Played by Wheat in Global Food Security. Food Secur..

[3] Zhao X., Fu X., Yin C., Lu F. (2021). Wheat Speciation and Adaptation: Perspectives from Reticulate Evolution. aBIOTECH.

[4] Sertse D., Fetene A., Leon J., You F.M., Cloutier S., McCartney C.A. (2024). Tracing Post-Domestication Historical Events and Screening Pre-Breeding Germplasm from Large Gene Pools in Wheat in the Absence of Phenotype Data. Theor. Appl. Genet..

[5] Yang F., Zhang J., Liu Q., Liu H., Zhou Y., Yang W., Ma W. (2022). Improvement and Re-Evolution of Tetraploid Wheat for Global Environmental Challenge and Diversity Consumption Demand. Int. J. Mol. Sci..

[6] Gituma J. (2023). Grain Central UK Farmer Sets World Records with Wheat, Barley Yields.

[7] Guinness World Records (2022). Guinness World Record Highest Wheat Yield.

[8] Ma S., Niu J., Si Y., Zheng S., Lu Y., Tian S., Shi X., Chen Z., Sun C., Qin Z. (2025). A Comprehensive Map of DNA-Segment Copy Number Variation in 491 Genomes of Common Wheat Uncovers Genes Associated with Multiple Agronomic Traits. Plant Commun..

[9] Knox A.K., Dhillon T., Cheng H., Tondelli A., Pecchioni N., Stockinger E.J. (2010). CBF Gene Copy Number Variation at Frost Resistance-2 Is Associated with Levels of Freezing Tolerance in Temperate-Climate Cereals. Theor. Appl. Genet..

[10] Boden S.A., McIntosh R.A., Uauy C., Krattinger S.G., Dubcovsky J., Rogers W.J., Xia X.C., Badaeva E.D., Bentley A.R., Brown-Guedira G. (2023). Updated Guidelines for Gene Nomenclature in Wheat. Theor. Appl. Genet..

[11] Dhillon T., Pearce S.P., Stockinger E.J., Distelfeld A., Li C., Knox A.K., Vashegyi I., Galiba G., Vágú A., Dubcovsky J. (2010). Regulation of Freezing Tolerance and Flowering in Temperate Cereals: The *VRN-1* Connection. Plant Physiol..

[12] Sertse D., Kassa M.T., McCallum B.D., von Wettberg E.J.B., McCartney C.A. (2025). Enhancing the Breeding Gene Pool of Wheat Using Accessions in Gene Banks as Demonstrated by the Watkins Collection. Theor. Appl. Genet..

[13] Yan L., Loukoianov A., Tranquilli G., Helguera M., Fahima T., Dubcovsky J. (2003). Positional Cloning of the Wheat Vernalization Gene *VRN1*. Proc. Natl. Acad. Sci. USA.

[14] Shitsukawa N., Ikari C., Mitsuya T., Sakiyama T., Ishikawa A., Takumi S., Murai K. (2007). Wheat SOC1 Functions Independently of *WAP1/VRN1*, an Integrator of Vernalization and Photoperiod Flowering Promotion Pathways. Physiol. Plant.

[15] Liu C.-J. (2022). Cytochrome B5: A Versatile Electron Carrier and Regulator for Plant Metabolism. Front. Plant Sci..

[16] Zhao X., Zhao Y., Gou M., Liu C.J. (2023). Tissue-Preferential Recruitment of Electron Transfer Chains for Cytochrome P450-Catalyzed Phenolic Biosynthesis. Sci. Adv..

[17] Noguchi M., Keino I., Takahashi H., Yamauchi S., Fujisawa M., Haga K., Sakai T., Takemiya A., Kodama Y. (2025). Phototropin 2 Mediates Daily Cold Priming to Promote Light Responses in Arabidopsis. J. Exp. Bot..

[18] Sakai T., Kagawa T., Kasahara M., Swartz T.E., Christie J.M., Briggs W.R., Wada M., Okada K. (2001). Arabidopsis Nph1 and Npl1: Blue Light Receptors That Mediate Both Phototropism and Chloroplast Relocation. Proc. Natl. Acad. Sci. USA.

[19] Seluzicki A., Chory J. (2025). Genetic Architecture of a Light-Temperature Coincidence Detector. Nat. Commun..

[20] Biniek C., Heyno E., Kruk J., Sparla F., Trost P., Krieger-Liszkay A. (2017). Role of the NAD(P)H Quinone Oxidoreductase NQR and the Cytochrome b AIR12 in Controlling Superoxide Generation at the Plasma Membrane. Planta.

[21] Zhou L., Ullah F., Zou J., Zeng X. (2025). Molecular and Physiological Responses of Plants That Enhance Cold Tolerance. Int. J. Mol. Sci..

[22] Filiz E., Kurt F. (2019). FIT (Fer-like Iron Deficiency-Induced Transcription Factor) in Plant Iron Homeostasis: Genome-Wide Identification and Bioinformatics Analyses. J. Plant Biochem. Biotechnol..

[23] Tripathi D.K., Singh S., Gaur S., Singh S., Yadav V., Liu S., Singh V.P., Sharma S., Srivastava P., Prasad S.M. (2018). Acquisition and Homeostasis of Iron in Higher Plants and Their Probable Role in Abiotic Stress Tolerance. Front. Environ. Sci..

[24] Qian Z., He L., Li F. (2024). Understanding Cold Stress Response Mechanisms in Plants: An Overview. Front. Plant Sci..

[25] Zolman B.K., Nyberg M., Bartel B. (2007). IBR3, a Novel Peroxisomal Acyl-CoA Dehydrogenase-like Protein Required for Indole-3-Butyric Acid Response. Plant Mol. Biol..

[26] Tao Y., Wu L., Volodymyr V., Hu P., Hu H., Li C. (2024). Identification of the Ribosomal Protein L18 (RPL18) Gene Family Reveals That TaRPL18-1 Positively Regulates Powdery Mildew Resistance in Wheat. Int. J. Biol. Macromol..

[27] Van Dieren A., Bittner A., Wurzinger B., Afjehi-Sadat L., Weckwerth W., Teige M., Vothknecht U.C. (2025). With or without a Ca^2+^ Signal? A Proteomics Approach towards Ca^2+^ Dependent and Independent Proteome Changes in Response to Oxidative Stress in *A. thaliana*. Planta.

[28] Jeon J., Cho C., Lee M.R., Van Binh N., Kim J. (2016). *CYTOKININ RESPONSE FACTOR2* (*CRF2*) and *CRF3* Regulate Lateral Root Development in Response to Cold Stress in Arabidopsis. Plant Cell.

[29] Lei L., Ding G., Cao L., Zhou J., Luo Y., Bai L., Xia T., Chen L., Wang J., Liu K. (2024). Genome-Wide Identification of CRF Gene Family Members in Four Rice Subspecies and Expression Analysis of OsCRF Members in Response to Cold Stress at Seedling Stage. Sci. Rep..

[30] Zwack P.J., Compton M.A., Adams C.I., Rashotte A.M. (2016). Cytokinin Response Factor 4 (CRF4) Is Induced by Cold and Involved in Freezing Tolerance. Plant Cell Rep..

[31] Myo T., Wei F., Zhang H., Hao J., Zhang B., Liu Z., Cao G., Tian B., Shi G. (2021). Genome-Wide Identification of the BASS Gene Family in Four Gossypium Species and Functional Characterization of GhBASSs against Salt Stress. Sci. Rep..

[32] Ji Z., Wang R., Zhang M., Chen L., Wang Y., Hui J., Hao S., Lv B., Jiang Q., Cao Y. (2024). Genome-Wide Identification and Expression Analysis of BrBASS Genes in Brassica Rapa Reveals Their Potential Roles in Abiotic Stress Tolerance. Curr. Issues Mol. Biol..

[33] Robinson D.G., Aniento F. (2020). A Model for ERD2 Function in Higher Plants. Front. Plant Sci..

[34] Zhu M., Fang Z., Wu Y., Dong F., Wang Y., Zheng F., Ma X., Ma S., He J., Liu X. (2024). A KDELR-Mediated ER-Retrieval System Modulates Mitochondrial Functions via the Unfolded Protein Response in Fission Yeast. J. Biol. Chem..

[35] Vorwerk S., Schiff C., Santamaria M., Koh S., Nishimura M., Vogel J., Somerville C., Somerville S. (2007). EDR2 Negatively Regulates Salicylic Acid-Based Defenses and Cell Death during Powdery Mildew Infections of Arabidopsis Thaliana. BMC Plant Biol..

[36] Kumar R., Tran L.-S.P., Neelakandan A.K., Nguyen H.T. (2012). Higher Plant Cytochrome B5 Polypeptides Modulate Fatty Acid Desaturation. PLoS ONE.

[37] Huang Y., Li C.Y., Biddle K.D., Gibson S.I. (2008). Identification, Cloning and Characterization of Sis7 and Sis10 Sugar-Insensitive Mutants of Arabidopsis. BMC Plant Biol..

[38] Wang H., Guo J. (2023). Molecular Cloning and Functional Characterization of Jatropha Curcas NCED3 Involved in Cold Resistance. Plant Biotechnol. Rep..

[39] Kalladan R., Lasky J.R., Sharma S., Kumar M.N., Juenger T.E., Des Marais D.L., Verslues P.E. (2019). Natural Variation in 9-Cis-Epoxycartenoid Dioxygenase 3 and Aba Accumulation. Plant Physiol..

[40] Diao P., Chen C., Zhang Y., Meng Q., Lv W., Ma N. (2020). The Role of NAC Transcription Factor in Plant Cold Response. Plant Signal Behav..

[41] Fang T., Wang Y., Chen H., Qu J., Xiao P., Wang Y., Jiang X., Li C., Liu J.-H. (2025). Genome-Wide Identification and Expression Profiles of NAC Transcription Factors in Poncirus Trifoliata Reveal Their Potential Roles in Cold Tolerance. BMC Plant Biol..

[42] Shi Y., Yang S. (2014). ABA Regulation of the Cold Stress Response in Plants. Abscisic Acid: Metabolism, Transport and Signaling.

[43] To J.P.C., Haberer G., Ferreira F.J., Deruère J., Mason M.G., Schaller G.E., Alonso J.M., Ecker J.R., Kieber J.J. (2004). Type-A Arabidopsis Response Regulators Are Partially Redundant Negative Regulators of Cytokinin Signaling. Plant Cell.

[44] Choi S., Prokchorchik M., Lee H., Gupta R., Lee Y., Chung E.-H., Cho B., Kim M.-S., Kim S.T., Sohn K.H. (2021). Direct Acetylation of a Conserved Threonine of RIN4 by the Bacterial Effector HopZ5 or AvrBsT Activates RPM1-Dependent Immunity in Arabidopsis. Mol. Plant.

[45] Toruño T.Y., Shen M., Coaker G., Mackey D. (2019). Regulated Disorder: Posttranslational Modifications Control the RIN4 Plant Immune Signaling Hub. Mol. Plant-Microbe Interact..

[46] Mandel M.A., Yanofsky M.F. (1998). The Arabidopsis AGL9 MADS Box Gene Is Expressed in Young Flower Primordia. Sex. Plant Reprod..

[47] Ma T., Wang S., Sun C., Tian J., Guo H., Cui S., Zhao H. (2023). Arabidopsis LFR, a SWI/SNF Complex Component, Interacts with ICE1 and Activates ICE1 and CBF3 Expression in Cold Acclimation. Front. Plant Sci..

[48] Tang X., Wang Q., Yuan H., Huang X. (2018). Chilling-Induced DNA Demethylation Is Associated with the Cold Tolerance of Hevea Brasiliensis. BMC Plant Biol..

[49] Li C., Guo Y., Wang L., Yan S. (2023). The SMC5/6 Complex Recruits the PAF1 Complex to Facilitate DNA Double-strand Break Repair in Arabidopsis. EMBO J..

[50] Maeyashiki C., Melhem H., Hering L., Baebler K., Cosin-Roger J., Schefer F., Weder B., Hausmann M., Scharl M., Rogler G. (2020). Activation of PH-Sensing Receptor OGR1 (GPR68) Induces ER Stress Via the IRE1α/JNK Pathway in an Intestinal Epithelial Cell Model. Sci. Rep..

[51] Yan L., Fu D., Li C., Blechl A., Tranquilli G., Bonafede M., Sanchez A., Valarik M., Yasuda S., Dubcovsky J. (2006). The Wheat and Barley Vernalization Gene *VRN3* Is an Orthologue of FT. Proc. Natl. Acad. Sci. USA.

[52] Chen A., Dubcovsky J. (2012). Wheat TILLING Mutants Show That the Vernalization Gene *VRN1* Down-Regulates the Flowering Re-pressor *VRN2* in Leaves but Is Not Essential for Flowering. PLOS Genet..

[53] Dubcovsky J., Loukoianov A., Fu D., Valarik M., Sanchez A., Yan L. (2006). Effect of Photoperiod on the Regulation of Wheat Vernalization Genes *VRN1* and *VRN2*. Plant Mol. Biol..

[54] Galiba G., Vágújfalvi A., Li C., Soltész A., Dubcovsky J. (2009). Regulatory Genes Involved in the Determination of Frost Tolerance in Temperate Cereals. Plant Sci..

[55] Sharma M., Marhava P. (2023). Regulation of PIN Polarity in Response to Abiotic Stress. Curr. Opin. Plant Biol..

[56] Pan J., Zhang M., Kong X., Xing X., Liu Y., Zhou Y., Liu Y., Sun L., Li D. (2012). ZmMPK17, a Novel Maize Group D MAP Kinase Gene, Is Involved in Multiple Stress Responses. Planta.

[57] Kumar K., Rao K.P., Sharma P., Sinha A.K. (2008). Differential Regulation of Rice Mitogen Activated Protein Kinase Kinase (MKK) by Abiotic Stress. Plant Physiol. Biochem..

[58] Kong X., Pan J., Zhang M., Xing X., Zhou Y., Liu Y., Li D., Li D. (2011). *ZmMKK4*, a Novel Group C Mitogen-Activated Protein Kinase Kinase in Maize (*Zea mays*), Confers Salt and Cold Tolerance in Transgenic *Arabidopsis*. Plant Cell Environ..

[59] Nitcher R., Pearce S., Tranquilli G., Zhang X., Dubcovsky J. (2014). Effect of the Hope FT-B1 Allele on Wheat Heading Time and Yield Components. J. Hered..

[60] Kitashova A., Schneider K., Fürtauer L., Schröder L., Scheibenbogen T., Fürtauer S., Nägele T. (2021). Impaired Chloroplast Positioning Affects Photosynthetic Capacity and Regulation of the Central Carbohydrate Metabolism during Cold Acclimation. Photosynth. Res..

[61] Nedo A.O., Liang H., Sriram J., Razzak M.A., Lee J., Kambhamettu C., Dinesh-Kumar S.P., Caplan J.L. (2024). CHUP1 Restricts Chloroplast Movement and Effector-triggered Immunity in Epidermal Cells. New Phytol..

[62] Arisz S.A., van Wijk R., Roels W., Zhu J.K., Haring M.A., Munnik T. (2013). Rapid Phosphatidic Acid Accumulation in Response to Low Temperature Stress in Arabidopsis Is Generated through Diacylglycerol Kinase. Front. Plant Sci..

[63] Kimura T., Jennings W., Epand R.M. (2016). Roles of Specific Lipid Species in the Cell and Their Molecular Mechanism. Prog. Lipid Res..

[64] Arisz S.A., Testerink C., Munnik T. (2009). Plant PA Signaling via Diacylglycerol Kinase. Biochim. Biophys. Acta Mol. Cell Biol. Lipids.

[65] Jia X., Si X., Jia Y., Zhang H., Tian S., Li W., Zhang K., Pan Y. (2021). Genomic Profiling and Expression Analysis of the Diacylglycerol Kinase Gene Family in Heterologous Hexaploid Wheat. PeerJ.

[66] Kim B.H., Kim S.Y., Nam K.H. (2012). Genes Encoding Plant-Specific Class III Peroxidases Are Responsible for Increased Cold Tolerance of the Brassinosteroid-Insensitive 1 Mutant. Mol. Cells.

[67] Yan J., Su P., Li W., Xiao G., Zhao Y., Ma X., Wang H., Nevo E., Kong L. (2019). Genome-Wide and Evolutionary Analysis of the Class III Peroxidase Gene Family in Wheat and *Aegilops tauschii* Reveals That Some Members Are Involved in Stress Responses. BMC Genom..

[68] Nakazawa M., Yabe N., Ichikawa T., Yamamoto Y.Y., Yoshizumi T., Hasunuma K., Matsui M. (2001). DFL1, an Auxin-Responsive GH3 Gene Homologue, Negatively Regulates Shoot Cell Elongation and Lateral Root Formation, and Positively Regulates the Light Response of Hypocotyl Length. Plant J..

[69] Jiang W., Yin J., Zhang H., He Y., Shuai S., Chen S., Cao S., Li W., Ma D., Chen H. (2020). Genome-Wide Identification, Characterization Analysis and Expression Profiling of Auxin-Responsive GH3 Family Genes in Wheat (*Triticum aestivum* L.). Mol. Biol. Rep..

[70] Niu J., Ma S., Zheng S., Zhang C., Lu Y., Si Y., Tian S., Shi X., Liu X., Naeem M.K. (2023). Whole-Genome Sequencing of Diverse Wheat Accessions Uncovers Genetic Changes during Modern Breeding in China and the United States. Plant Cell.

[71] Cao Q., Zhu Z., Xu D., Wu J., Xu X., Dong Y., Bian Y., Ding F., Zhao D., Tu Y. (2024). Characterization of a 4.1 Mb Inversion Harboring the Stripe Rust Resistance Gene YR86 on Wheat Chromosome 2AL. Crop J..

[72] Zhu Z., Cao Q., Han D., Wu J., Wu L., Tong J., Xu X., Yan J., Zhang Y., Xu K. (2023). Molecular Characterization and Validation of Adult-Plant Stripe Rust Resistance Gene Yr86 in Chinese Wheat Cultivar Zhongmai 895. Theor. Appl. Genet..

[73] Zhuang L., Liu H., Hou J., Jian C., Liu Y., Li H., Xi W., Zhao J., Hao P., Liu S. (2024). Genetic Improvement of Important Agronomic Traits in Chinese Wheat Breeding over the Past 70 Years. BMC Plant Biol..

[74] Zhang X., Liu X., Wang L., Zhao Q., Yu Y., Du R., Xu Y., Zhen W., Wang Y. (2024). Wheat Yield and Grain-Filling Characteristics Due to Cultivar Replacement in the Haihe Plain in China. Front. Plant Sci..

[75] Luo Q., Zheng Q., Tong C., Jia H., Liu L., Yin M., Xie J., Li H., Wang H., Chen Z. (2025). The Location and Genome Origin of Alien Chromatin in Wheat Founder Parent Xiaoyan 6. Theor. Appl. Genet..

[76] Jung W.J., Jeong J.H., Yoon J.S., Seo Y.W. (2025). Investigation of Wheat Cold Response Pathway Regulated by TaICE41 and TaCBFIVd-B9 through Brachypodium Distachyon Transformation. Plant Sci..

[77] Schulthess A.W., Kale S.M., Zhao Y., Gogna A., Rembe M., Philipp N., Liu F., Beukert U., Serfling A., Himmelbach A. (2022). Large-Scale Genotyping and Phenotyping of a Worldwide Winter Wheat Genebank for Its Use in Pre-Breeding. Sci. Data.

[78] Cheng S., Feng C., Wingen L.U., Cheng H., Riche A.B., Jiang M., Leverington-Waite M., Huang Z., Collier S., Orford S. (2024). Harnessing Landrace Diversity Empowers Wheat Breeding. Nature.

[79] Luu K., Bazin E., Blum M.G.B. (2017). *Pcadapt*: An R Package to Perform Genome Scans for Selection Based on Principal Component Analysis. Mol. Ecol. Resour..

[80] Dong S.S., He W.M., Ji J.J., Zhang C., Guo Y., Yang T.L. (2021). LDBlockShow: A fast and convenient tool for visualizing linkage disequilibrium and haplotype blocks based on variant call format files. Brief. Bioinform..

[81] Pebesma E. (2018). Simple Features for R: Standardized Support for Spatial Vector Data. R J..

