# Supplementary figures and images for: Genomic Insights into Winter Wheat Breeding for Severely Cold Climates
Source: Int J Mol Sci. 2026 Feb 5;27(3):1568. doi: 10.3390/ijms27031568 (PMC12898387; doi:10.3390/ijms27031568)

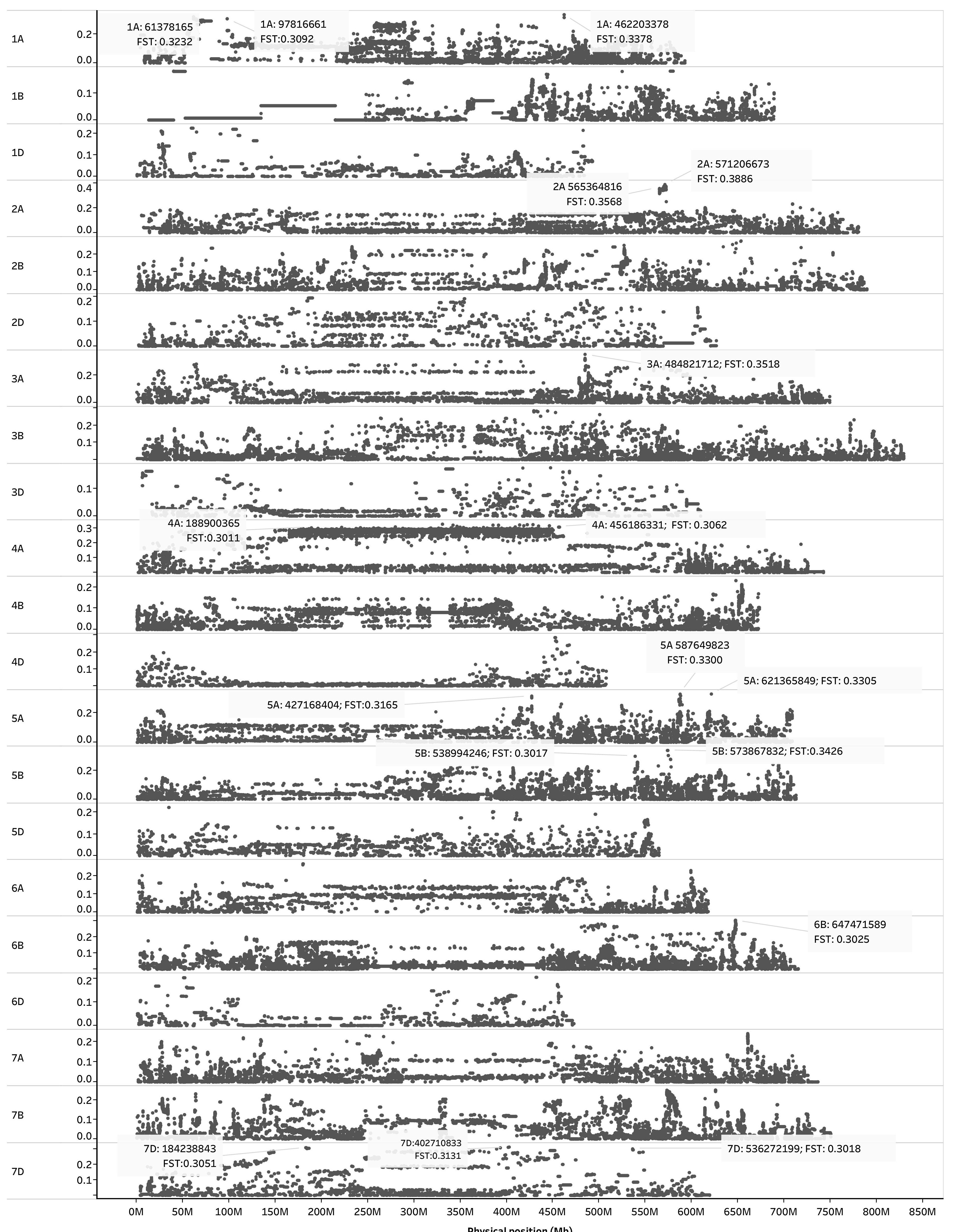

Supplement: Supplementary file 1 [file ijms-27-01568-s001.zip › ijms-4045190-Figure S1.jpg]

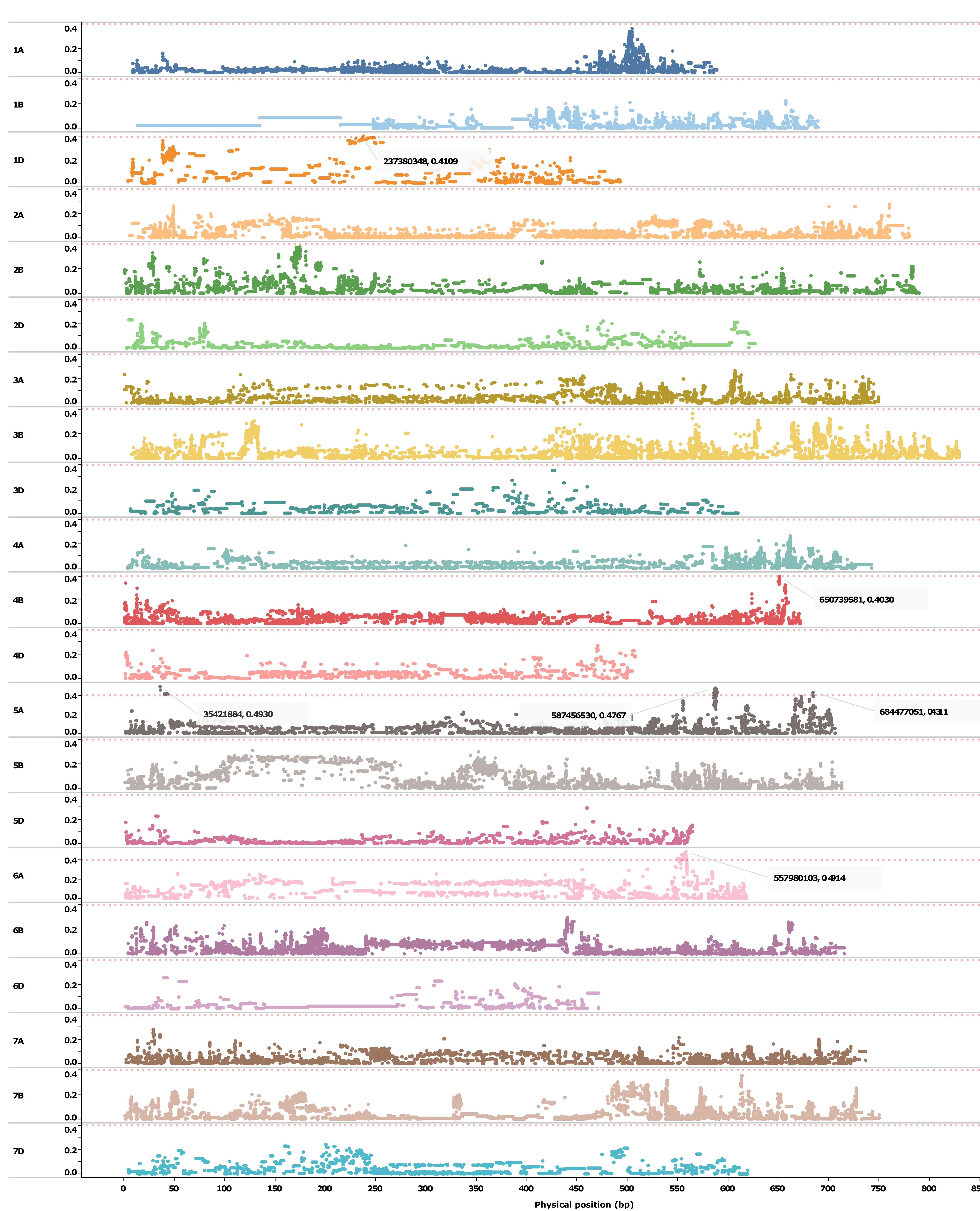

Supplement: Supplementary file 1 [file ijms-27-01568-s001.zip › ijms-4045190-Figure S2.jpg]
